# Diverse Presentation of Breath Holding Spells: Two Case Reports with Literature Review

**DOI:** 10.1155/2013/603190

**Published:** 2013-09-26

**Authors:** Geetanjali Rathore, Paul Larsen, Cristina Fernandez, Manish Parakh

**Affiliations:** ^1^Pediatric Neurology, Baylor College of Medicine, Houston, TX 77030, USA; ^2^Pediatric Neurology, University of Nebraska Medical Center, Omaha, NE 68198, USA; ^3^Pediatrics, Creighton University, Omaha, NE 68131, USA; ^4^Pediatric Neurology, Umaid Women and Children's Hospital, Jodhpur, Rajasthan 342001, India

## Abstract

Breath holding spells are a common and dramatic form of syncope and anoxic seizure in infancy. They are usually triggered by an emotional stimuli or minor trauma. Based on the color change, they are classified into 3 types, cyanotic, pallid, and mixed. Pallid breath holding spells result from exaggerated, vagally-mediated cardiac inhibition, whereas the more common, cyanotic breathholding spells are of more complex pathogenesis which is not completely understood. A detailed and accurate history is the mainstay of diagnosis. An EKG should be strongly considered to rule out long QT syndrome. Spontaneous resolution of breath-holding spells is usually seen, without any adverse developmental and intellectual sequelae. Rare cases of status epilepticus, prolonged asystole, and sudden death have been reported. Reassurance and education is the mainstay of therapy. Occasionally, pharmacologic intervention with iron, piracetam; atropine may be of benefit. Here we present 2 cases, one of each, pallid and cyanotic breath holding spells.

## 1. Introduction

Breath holding spells are paroxysmal nonepileptic events of infancy. These were first described in 1737 by Nicholas Culpepper and were thought to be voluntary breath holding. These episodes are often precipitated by emotional stimuli like anger, frustration, sudden fright, or minor trauma [1]. The child cries vigorously, followed by end-expiration apnea, leading to cyanosis or pallor, loss of consciousness, and occasionally convulsive movements. Even though these episodes are benign, they can be rather frightening for parents and others observing a spell. Serious complications of breath holding spells are rare, but cases of sudden death, prolonged asystole, and status epilepticus have been reported. A detailed history and exam are important to diagnose theses spells and help distinguish from epileptic seizures and other causes of syncope. The cornerstone of therapy is reassuring the parents and educating them about the condition. Iron therapy, piracetam, levetiracetam, and atropine are considered as treatment and have shown variable efficacy.

## 2. Presentation

### 2.1. Case 1

 A 38-day-old female was seen in clinic for frequent episodes of cyanosis and one episode of possible multifocal clonic seizure. She was born term via repeat caesarean section, needed stimulation, and supplemental oxygen after birth for 24 hours. She received 72 hours of antibiotics in the NICU and discharged home on room air. At the 6th day of life, about 10 minutes after breast feeding, she was laying supine when helding her breath, got cyanotic, and had clonic jerking of all 4 extremities. This lasted about 1 minute and aborted on turning her semiprone. After the event she had generalized hypotonia and fell asleep. She was admitted to the NICU where her CBC, CMP, ionized calcium, magnesium, phosphorous, CSF, EEG, ABR, MRI brain, and EKG were all within normal limits. She was discharged on PHB. Four days prior to presenting in clinic, she had a similar episode after a bath and also on the next two following days. She is the 4th issue of a 28-year-old mother who has history of a 1st and 2nd trimester miscarriage followed by an intrauterine demise after failure to progress. Family history is significant only for a maternal first cousin with a history of BHS and seizures until 2-years-old, now a 16-year-old doing well. Her physical exam was unremarkable including a normal neurologic and cardiologic exam. She was started on levetiracetam and weaned off phenobarbital. On followup 2 months later she was developing well, still having a few episodes but reduced in frequency. 

### 2.2. Case 2

A 17-month-old male was seen in clinic for evaluation of spells. His first episode was 6 months prior to presentation when he slipped at the poolside and hit the back of his head. He had no loss of consciousness and got up on his own, looked pale and loopy, and passed out. Since then he has had 3 similar episodes where he has tripped or fallen, with or without bumping his head, he will stand up, look pale, and then pass out. He has had limb stiffening and his eyes seem to roll up. No apnea, jerking, and incontinence are associated with these episodes. He is otherwise a healthy boy with past history only significant for eczema. Family history is unremarkable. He did get a CT scan after his first episode, which was normal. An EEG was not performed as these were thought to be consistent with pallid form of breath holding spells. An EKG was performed to rule out cardiac etiology and CBC to rule out IDA. 

## 3. Discussion

Breath holding spells are a common nonepileptic paroxysmal disorder of infancy. The term breath holding spells, however, is a misnomer, as these episodes are involuntary and occur during expiration. Breath holding spells have an incidence of 4.6% to 4.7% [1, 2], with a male to female ratio of 3 : 1 [3]. A positive family history is present in 23% to 38% of children with spells. The inheritance pattern based on a regression model for pedigree analysis suggests autosomal dominant pattern with reduced penetrance [4]. The typical age of onset is between 6 months to 18 months [5]. Occasionally the onset may occur in the first few weeks of life, with up to 15% of children having onset of spells at less than 6 months of age [5–8]. Less than 10% have onset after 2 years [7]. The frequency can range from several spells a day to once a year, with the majority having 1–6 spells a week [7]. The spells tend to decrease in frequency during second year of life, by 4 years 50% will have complete resolution of spells, and by age of 8 years almost all children stop having any spells [6, 8].

Breath holding spells are classified based on the color change manifested by the child during the event in cyanotic, pallid, or mixed episodes. Cyanotic episodes are more common, seen in 54–62%, whereas pallid and mixed types are seen in about 19–24% each [1]. 

Cyanotic breath holding spells are precipitated by emotional stimuli like anger or frustration which causes the child to cry vigorously then become silent and hold the breath in expiration. This is followed by rapid onset of cyanosis and may resolve at this point by return to regular breathing or may progress onto loss of consciousness, limpness, or even opisthotonic posturing [9]. Pallid breath holding spells on the other hand are usually provoked by sudden fright or pain. A fall or minor injury to the head is often the precipitating event. Child may gasp or cry briefly then becomes quiet and pale and loses consciousness. More severe spells may progress to clonic jerking and incontinence. Serious complications of breath holding spells are rare. However, breath holding spells associated with asystole have been reported to cause sudden death [10]. In other reported deaths in patients with breath holding spells, death was caused due to other underlying conditions [11, 12]. In rare cases, breath holding spells have been followed by status epilepticus [13–15].

The pathophysiology of cyanotic breath holding spells is not very well understood. Intrapulmonary shunting [12], disorder of ventilator chemosensitivity [16], and autonomic dysregulation [17–19] are the postulated theories. Pallid breath holding spells are known to be caused by exaggerated vagal response leading to cerebral hypoperfusion [20]. Ocular compression can trigger these spells via the oculocardiac reflex which increases vagal tone. About 70% of the patients with pallid spells go into vagally mediated asystole on ocular compression [1]. Iron deficiency anemia has also been shown to play a role in the pathophysiology of breath holding spells [3, 21, 22]. A study showed complete resolution of spells in 50% patients on iron therapy and 50% reduction in another 36.4% [23]. A recent study has also suggested a possible relationship between maternal iron deficiency anemia and children with breath holding spells [24]. Iron's role is thought to be due to it being a cofactor in catecholamine metabolism and neurotransmitter function [23].

An accurate and detailed history of the events is used to make the diagnosis and usually no laboratory tests are needed. An EEG is not recommended unless there is history of prolonged seizure or the possibility of an epileptic seizure which cannot be ruled out on basis of history alone. Long QT syndrome was though rare but can present similar to breath holding spells; thus an EKG should be considered in all patients with breath holding spells [9, 24].

The mainstay of therapy is reassurance and education. The parents should be reassured of the benign nature of these spells with a normal developmental and intellectual outcome [25]. The parents and care takers should be instructed to place the child in a lateral recumbent position during the breath holding spell to reduce the period of cerebral anoxia. Iron therapy should be initiated in children with iron deficiency anemia. It should also be considered in children without anemia as iron therapy has showed benefit in this group also [23]. As the convulsive episodes are nonepileptic, anticonvulsants are not recommended. Rarely atropine may be considered in children with pallid breath holding spells [26]. There have been cases of prolonged asystole with breath holding spells warranting the placement of a permanent cardiac pacemaker [27, 28]. Piracetam has been shown to be safe and effective in treating BHS [29]. In one study, resolution of spells after 2-month therapy with piracetam, was seen in 92% children, compared to 30% resolution in the placebo group [30]. This drug is approved for treatment of myoclonus but not FDA approved for breath holding spells. Glycopyrrolate, theophylline, fluoxetine, and levetiaciteram have been used to treat breath holding spells in individual cases and need further studies to confirm efficacy and safety [31–33].

 In summary, breath holding spells are common and dramatic events in childhood, rarely being life threatening. Mainstay of management is education and reassurance. Iron therapy and piracetam have shown to be beneficial. In severe forms, specially associated with prolonged asystole or seizure, pharmacological and even surgical measures may be indicated.
